# MARCKS as a mediator and target for pathological tunneling nanotubes between patient-derived glioblastoma cells and astrocytes

**DOI:** 10.1038/s41419-026-09109-x

**Published:** 2026-07-13

**Authors:** Lauren C. Nassour-Caswell, Mayada Ahmed, Shane T. Rich-New, Taylor L. Schanel, Manoj Kumar, Nicholas J. Eustace, Timothy D. Rohrbach, John S. Jarboe, Patricia H. Hicks, Joshua C. Anderson, Christopher D. Willey

**Affiliations:** 1 Department of Radiation Oncology, Heersink School of Medicine, University of Alabama at Birmingham, Birmingham, AL USA; 2 Department of Biochemistry and Molecular Biology, University of Alabama at Birmingham, Birmingham, AL USA; 3 Department of Radiation Oncology, City of Hope National Medical Center, Duarte, CA USA; 4 Department of Natural Sciences and Mathematics, Richard Bland College, South Prince George, VA USA; 5 Central Alabama Radiation Oncology, Montgomery, AL USA

**Keywords:** CNS cancer, Cancer microenvironment

## Abstract

During glioblastoma (GBM) progression, therapeutic resistance is influenced by a heterogeneous network of tumor- and tumor-promoting subpopulations within the tumor microenvironment. Therapeutic interventions against tumor cells, such as chemoradiotherapy, can further induce adaptive defense mechanisms mediated by sophisticated intercellular communication and aberrant signaling pathways. Tunneling nanotubes (TNTs) have emerged as key mediators of this process, promoting tumor cell survival through metabolic rescue while enabling the recruitment and reprogramming of surrounding normal cells into tumor-supportive phenotypes. This dynamic is exemplified in GBM, where TNT-mediated interactions between brain tumor–initiating cells (BTICs) and normal human astrocytes (NHAs) highlight the need to elucidate the molecular mediators underlying this crosstalk. Myristoylated Alanine Rich C-Kinase Substrate (MARCKS) has never been investigated as a potential regulator of TNTs despite several overlapping signaling pathways and its influence on GBM therapeutic resistance. In the present study, we demonstrate a role for the MARCKS effector domain (ED) and PKC activation in the formation and functionality of TNTs between PTEN-null GBM BTICs and NHAs. We employ a MARCKS phosphorylation site (MPS) peptide derived from MARCKS effector domain (MED2), PKC-targeting drugs, and an inducible MARCKS ED U87 model to elucidate a potential role for MARCKS phosphorylation and PKC in TNT regulation between GBM cells (i.e., BTICs or U87s) and NHAs.

## Introduction

The development of therapeutic resistance in glioblastoma (GBM) remains a significant obstacle to improving patient outcomes and increasing overall survival times. GBM tumors form interconnected networks comprised of heterogeneous populations of tumor and non-tumor cells, which create a resilient tumor microenvironment (TME) that promotes the development of treatment resistance and dissemination from the origin site [1–5]. Rapid intercellular communication and organelle exchange within these networks support this by facilitating metabolic rescue of tumor cells and infiltration of surrounding brain tissue [4, 6–8]. However, the molecular mediators driving these communication pathways remain underexplored and represent potential therapeutic targets.

Direct cellular communication can occur through tumor microtubes (TMs) predominantly found between tumor cells, and tunneling nanotubes (TNTs), which are universal transport conduits between diverse cell types [4, 6, 8–10]. Tunneling nanotubes were first identified in rat pheochromocytoma PC12 cells by the Rustom et al. group and were shown to also be an in vivo phenomenon shortly after [11–13]. Since their discovery, TNTs have been identified in cellular communication and treatment resistance across several diseases, including GBM [3–6, 8, 9]. While TNTs originate from normal physiological events such as embryonic development, their prevalence decreases with age. In adults, increased TNTs are associated with metabolic stress [3, 4, 6, 8, 10, 14–16]. Under such conditions, TNTs can mediate the transfer of mitochondria from healthy cells to damaged ones, promoting cell survival, such as a neuron during an ischemic stroke [16–21]. In GBM, this same rescue mechanism is hijacked, allowing rapidly proliferating cells to enhance their own survival under oxygen-limited conditions.

Astrocytes are ideal participants in this process due to their abundance and engagement in normal brain processes. GBM cells plunder mitochondria from tumor-supportive astrocytes by exploiting normal processes [16–19, 22, 23]. GBM cells can also transfer damaged mitochondria with mutated mitochondrial DNA (mtDNA) to surrounding NHAs, reprogramming them into tumor-associated astrocytes (TAA) that enhance therapeutic resistance [3, 4, 9, 17, 19]. Several groups have shown TAA’s can decrease GBM sensitivity to temozolomide (TMZ) and radiotherapy, enhance their invasive capacity, and recruit other cells to the TME in a TNT-mediated manner [22, 24–26]. Although TNT formation is normally tightly regulated, aberrant signaling in GBM drives pathological TNT activity, highlighting the need to identify key regulatory mechanisms [4, 6–8].

TNTs are rich in filamentous actin (F-actin), and actin-modulating dynamics and associated pathways are critical to consider. The PI3K–AKT–mTOR pathway is a known driver of TNTs and is among the most frequently hyperactivated pathways in GBM [4, 6, 8–10, 14]. Phosphorylation of the MARCKS ED, primarily by protein kinase C (PKC) or other kinases induces cytosolic translocation of MARCKS and phosphatidylinositol 4,5-bisphosphate (PIP_2_) release [27–30]. This enables activation of phosphoinositide 3-kinase (PI3K) and downstream mediators such as protein kinase B (AKT) and mammalian target of rapamycin (mTOR), resulting in increased TNT formation, proliferation, migration, and resistance [31–33]. The PI3K pathway is negatively regulated by the tumor suppressor, phosphatase and tensin homolog (PTEN); however, 40% of GBM cases present a loss of PTEN function [31, 34–37]. PTEN loss promotes injury-like responses, self-renewal, resistance to temozolomide (TMZ), and processes associated with TNT utilization [8, 38–40]. In PTEN-deficient cells, PIP_2_ availability becomes dependent on Myristoylated Alanine-Rich C Kinase Substrate (MARCKS), though its role in TNT dynamics has not been explored [35–37].

MARCKS phosphorylation is implicated in GBM therapeutic resistance [30], and is known for its regulation of polarized signaling networks and the cytoskeleton through actin modulation, mediated through its electrostatically charged effector domain (ED) [27, 28, 34, 41–44]. We have shown previously that U87 lines modified to overexpress a pseudo-phosphorylated MARCKS ED exhibit increased radioresistance compared to those that overexpress a non-phosphorylated variant [28, 45]. Moreover, PKC is highly influential in MARCKS ED phosphorylation and has been implicated in adrenocorticoid carcinoma cell TNT formation, but its role in GBM-TNTs has not been reported [46]. We previously demonstrated that a MARCKS phosphorylation site (MPS) peptide derived from MARCKS ED (MED2) induces cytotoxicity and retraction of cytoplasmic extensions in GBM patient-derived tumor-initiating cells [42]. Given the membrane-derived nature of TNTs and the central role of MARCKS phosphorylation events on the PI3K-AKT-mTOR pathway in the absence of PTEN, we hypothesized that MARCKS phosphorylation and upstream PKC activity regulate aberrant TNT function in GBM, and this process can potentially be targeted using the MED2 peptide.

Using a TNT-promoting co-culture system of PTEN-null GBM cells and normal human astrocytes [47], we show that PKC activation increases TNT formation and mitochondrial transfer, while PKC inhibition or MED2 treatment produces opposing effects. Furthermore, NHA co-cultures with inducible U87 models overexpressing wild-type, pseudo-phosphorylated, or non-phosphorylated [30, 48] MARCKS ED exhibit distinct TNT phenotypes that phenocopy PKC manipulation. Together, these findings identify PKC and MARCKS as novel regulators of TNT dynamics mediating astrocyte–GBM communication.

## Results

### Astrocytes increase TMZ resistance in JX14

**Fig. 1:**
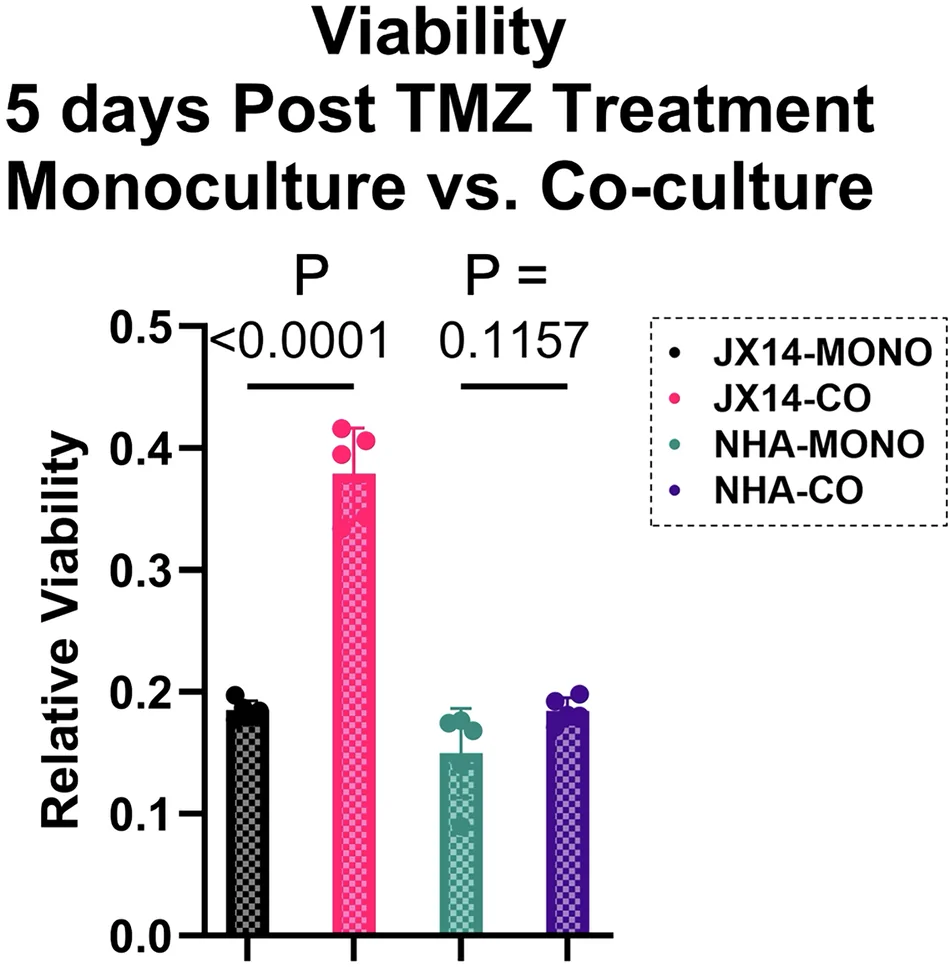
Astrocytes increase TMZ resistance in JX14. Viability of TMZ-treated JX14 cells and NHAs five days post-treatment. Cells were tested in homogeneous (monoculture) and heterogeneous (co-culture) conditions separated by a transwell insert. JX14 cells indirectly cultured with NHAs exhibited a two-fold increase in viability compared to those in homogenous culture. ****p < 0.0001 (JX14-co vs. Jx14-mono, actual *p* < 0.0001) by one-way ANOVA with Šidák’s test. NHAs exhibited no difference in viability in either condition. *N* = 5 replicates analyzed per experimental condition, repeated in three independent cultures. All graph error bars represent SEM.

JX14 cells naturally possess an epigenetically silenced, unmethylated MGMT promoter and are PTEN-null. Previous studies have reported that although JX14 has an unmethylated MGMT promoter, it is partially sensitive to TMZ [49]. We investigated that JX14 co-cultured with NHAs exhibited increased resistance to TMZ. We began culturing JX14 and NHAs as monocultures and indirect co-cultures designed to favor TNT-mediated events to assess for cytotoxicity after 5 days. NHA monocultures and co-cultures were both sensitive to TMZ when cultured alone, resulting in 15% viability $\pm \,0.0366)$ and 18.5% viability $\pm \,0.0109)$ respectively (*P* = 0.1157) (Fig. 1A). Monocultured JX14 resulted in 18.5% viability $\pm \,0.0078)$ following TMZ treatment, however, JX14 demonstrated a survival advantage when exposed to NHAs, with increased viability of 37.9% $\pm \,0.0376)$ (*P* < 0.0001) (Fig. 1A). Our next goal was to determine if our protein of interest was present in TNT structures.

### MARCKS is present in functional TNTs between JX14 and NHAs

**Fig. 2:**
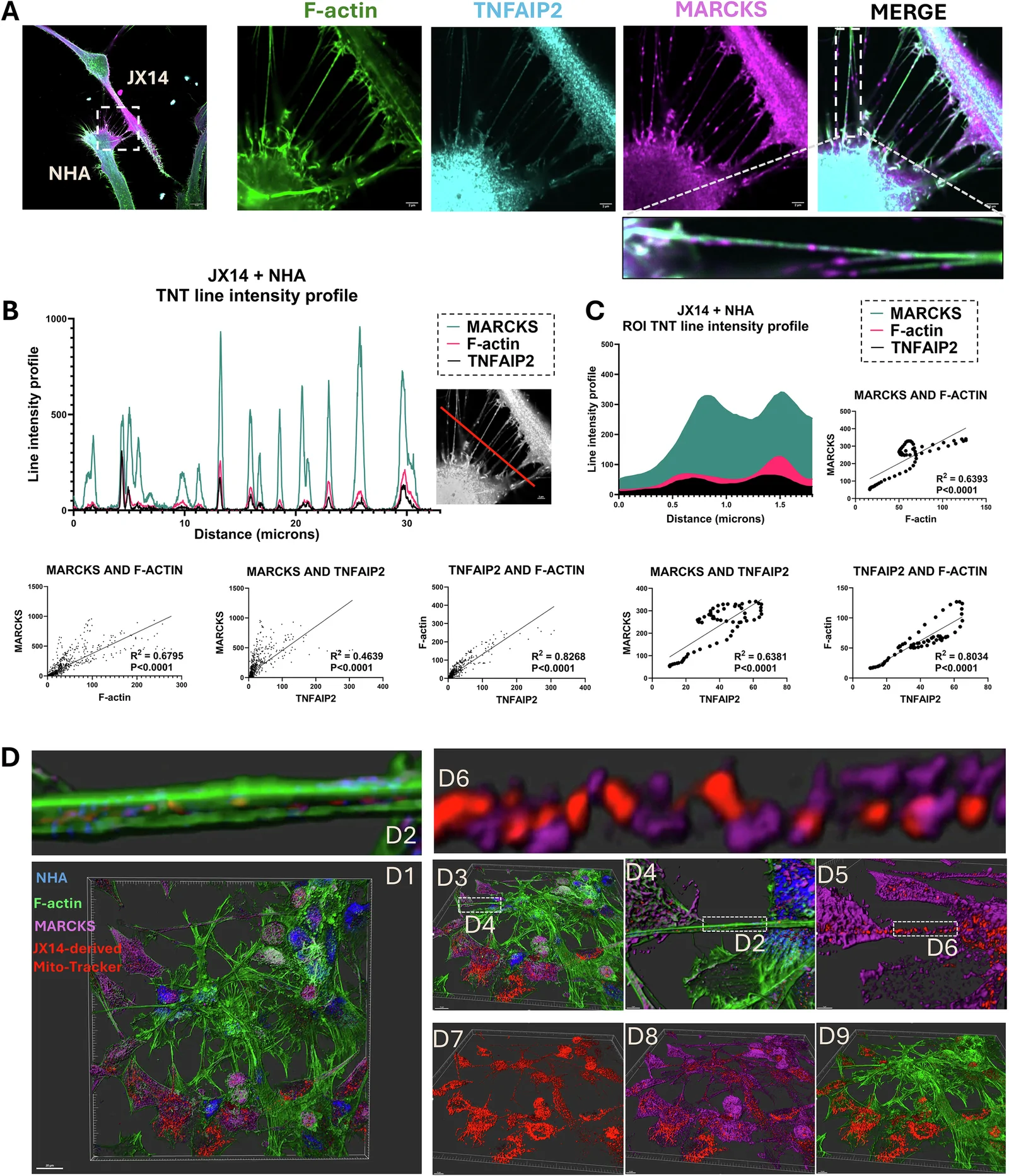
MARCKS localizes in Heterotypic GBM-Astrocyte TNTs. **A** Representative maximum-intensity widefield fluorescent images of GBM PDX-derived JX14 and NHAs incubated for 18 h, probed with anti-MARCKS, phalloidin, and anti-TNFAIP2. Left panel shows a merged view of NHAs and JX14, indicated with a 20 µm scale bar. The Image set on the right shows individual fluorescent channels with indicated antibody targets and 2 µm scale bars. ROI shows a close view of tri-color merge of single TNT (white box, 2 µm inset). **B** Correlation of target proteins shown through line intensity profile plot (red line across TNTs on grayscale inset). ****p < 0.0001 (MARCKS vs F-actin: R^2^ = 0.6795, actual *p* < 0.0001; MARCKS vs TNFAIP2: R^2^ = 0.4639, actual *p* < 0.0001; TNFAIP2 vs F-actin: R^2^ = 0.8268, actual *p* < 0.0001) by Pearson’s Correlation with simple linear regression. **C** Correlation metrics of target proteins and F-actin in a single TNT ROI. ****p < 0.0001 (MARCKS vs F-actin: R^2^ = 0.6393, actual *p* < 0.0001; MARCKS vs TNFAIP2: R^2^ = 0.6381, actual p < 0.0001; TNFAIP2 vs F-actin: R^2^ = 0.8034, actual *p* < 0.0001) by Pearson’s Correlation with simple linear regression. **D** Imaris-rendered 3D still-image depictions of Supplementary video SV1. Various views of NHA (Labeled with CellTrackerBlue) co-cultured with JX14 (labeled with MitoTrackerRed) probed for anti-MARCKS (magenta) and F-actin (green), showing TNT-connection in 3D-rendered wide view (D1, D3, D7-D9), TNT zoom (D4 and D5), and TNT-ROI zoom (D2 and D6).

To determine if MARCKS is involved in TNT dynamics, we performed immunofluorescence on NHAs co-cultured with JX14 cells for 18 h at a low cell density. We identified MARCKS in heterotypic TNT-like structures that stained positive for F-actin and the TNT marker, TNFα-induced protein 2 (TNFAIP2) (Fig. 2A). Next, we sought to determine the degree of overlap of these proteins by plotting the line intensity profile across the numerous TNT structures present. Pearson’s correlation revealed positive correlations amongst all proteins (Fig. 2B). To increase specificity, we performed the same analysis on a single TNT-ROI, for which we observed similar positive correlation trends across all proteins (Fig. 2C). To determine whether the TNTs were functional, we assessed mitochondrial transfer within heterotypic TNTs by staining JX14 cells with MitoTracker Red and labeling NHAs with a distinct whole-cell tracker blue dye. The two cell types were then co-incubated at a higher cell density for 3.5 h to facilitate TNT formation and potential mitochondrial exchange. Using high-resolution confocal microscopy, we identified mitochondria within a clearly defined TNT structure (Fig. 2D, subpanel D2). Using 3D-reconstruction of Z-stacked images and processing in Imaris, we visualized MARCKS co-localizing with mitochondria in the TNT (Fig. 2D, subpanel D6). A MitoTracker map overlayed with anti-MARCKS and phalloidin staining suggests a close relationship between TNT transport and MARCKS expression (Fig. 2D, subpanel D7-9). A distinct feature that distinguishes TNTs from filopodia is their suspended nature above the substratum. We utilized Imaris 3D processing to demonstrate this (Supplementary video SV1).

### MARCKS effector domain peptide alters TNT formation and functionality

**Fig. 3:**
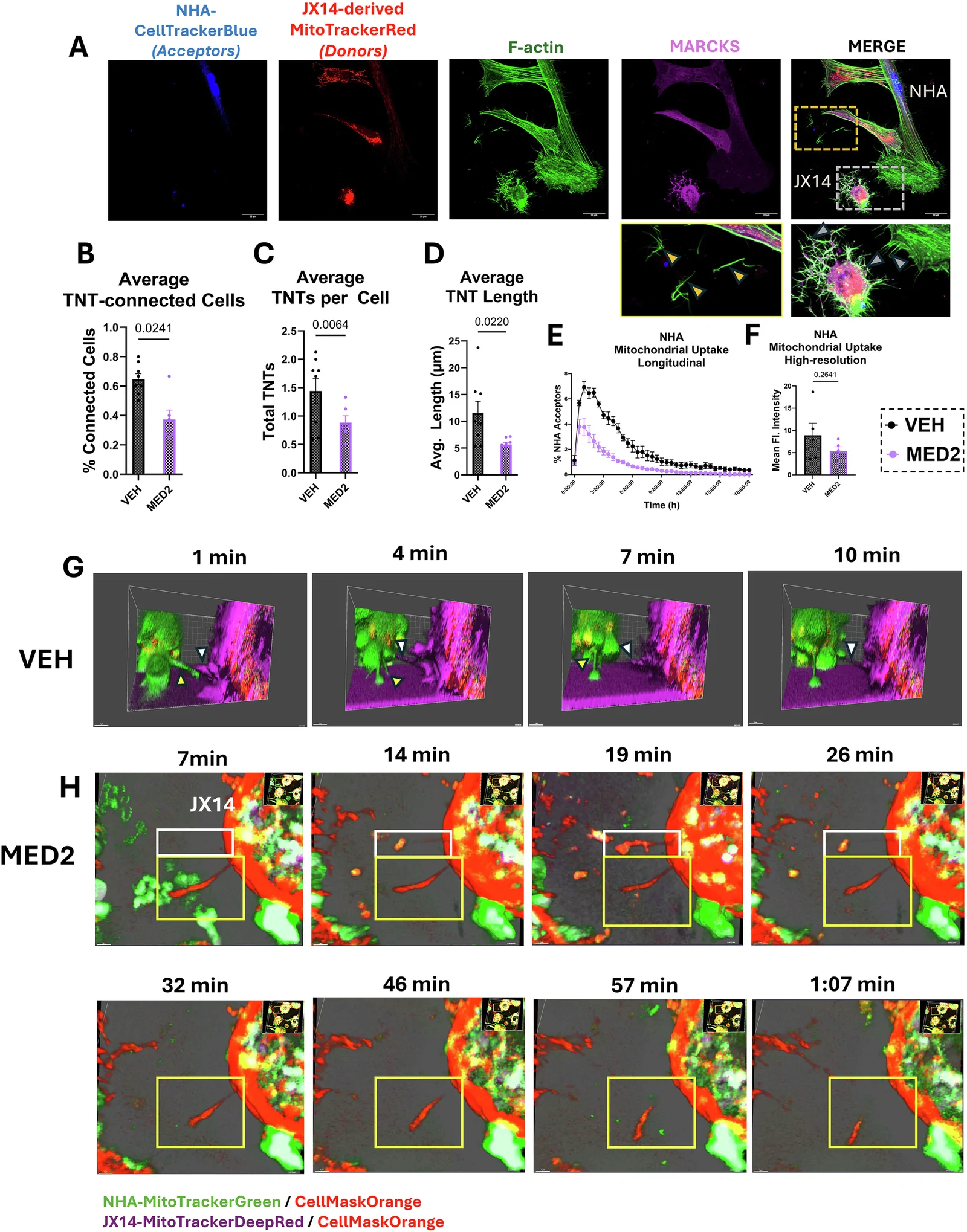
MARCKS effector domain peptide alters TNT mechanics and functionality. **A** Representative maximum-intensity widefield fluorescent images of JX14 co-cultured with NHAs, followed by treatment with 3 µM MED2 for 4 h. ROIs show distorted TNT morphology (white box, white arrows) and TNT-like fragments shed into the extracellular space (orange box, orange arrows), 20 µm scale bar. **B** Number of cells connected by TNTs decreases under MED2 conditions *p < 0.05 (VEH vs MED2, actual *p* = 0.0241) by unpaired Student’s *t*. C Average TNTs per cell quantified between NHAs and JX14 were decreased under MED2 test conditions compared to VEH **p < 0.01 (VEH vs MED2, actual *p* = 0.0064) by unpaired Student’s *t* test. **D** Average length of TNTs between JX14 and NHAs exhibited a decrease compared to control *p < 0.05 (VEH vs MED2, actual *p* = 0.0220) by unpaired Student’s *t* test. **E** Percentage of NHAs with JX14-derived mitochondria quantified longitudinally every 30 min over 18 h *p < 0.05 (2 h peak, VEH vs MED2, actual *p* = 0.0247) by two-way ANOVA with repeated measures and Šidák’s comparison. **F** Mean fluorescent intensity of NHA mitochondrial confocal imaging at 3.5 h. **G** Live cell kinetic confocal imaging depicting TNT formation, extension, and retraction between JX14 (magenta, white arrows) and NHA (green, yellow arrows), as shown in Supplementary video SV2. 2 µm scale bar. **H** Live cell kinetic confocal imaging close-up of MED-treated JX14 TNT formation and extension (white box) and TNT extension attempt (yellow box). TNT-like extension tethered to JX14 appears to fail extension and pinch off into the extracellular space (yellow box), as shown in Supplementary video SV3. 1 µm scale bar. TNT metrics, *N* = 8 VEH, *N* = 6 MED2 were analyzed per experimental condition out of 3 independent cultures. Mitochondrial transfer, *N* = 5 were analyzed per experimental condition out of three independent cultures. All graph error bars represent SEM.

Our aforementioned MED2, generated by conjugating the MARCKS ED to a cell-penetrating HIV-TAT sequence, induced the retraction of cellular extensions in BTIC xenoline, XD456, when adhered to Geltrex [42]. Due to the differential PTEN transcription profiles of XD456 (PTEN-WT) and JX14 (PTEN-null), we aimed to determine if this same result occurred in JX14. We observed retraction of cellular membrane extensions in JX14 infected with mCherry lentivirus upon exposure to 3 µM MED2 for 1.5 h in 30-min increments (data not shown). Next, we aimed to determine how MED2 affected the integrity and function of heterotypic TNTs between JX14 and NHAs. We noted a decrease in TNTs and abnormal structures in any TNT-like structures (Fig. 3A, gray box/arrows). TNT-like fragments were released into the extracellular space (Fig. 3A, orange box/arrows). MED2-treated cells exhibited decreased average TNT-connected cells, TNTs per cell, and TNT length compared to the vehicle control (Fig. 3B–D). To investigate functionality, mitochondrial transfer in direct contact was quantified based on Mito Tracker Red accumulation in acceptor cells, which is dependent on membrane potential. MED2 treatment reduced NHAs uptake of JX14-derived mitochondria and NHAs’ mitochondrial intensity (Fig. 3E, F). Next, we aimed to determine the effects of MED2 on transient TNTs in real-time using live cell confocal imaging (Supplementary Video SV2). We observed that TNTs readily form between JX14 and NHAs under normal conditions (Fig. 3G). Extension of the TNT, contact with the adjacent cell, and retraction into the originating cell occur over a short period of approximately six min, with each event occurring roughly three min apart (Fig. 3G, white and orange arrows). Upon addition of MED2, the time to form, extend, and retract a TNT increased to approximately 12 min, indicating MED2 might slow the TNT formation process (Fig. 3H, white box, Supplementary Video SV3). Furthermore, the nascent TNT exhibited impaired structural integrity, failing to maintain the characteristic linear morphology during extension. An adjacent TNT (tethered to the membrane) was pinched off (Fig. 3E, yellow box), and we suspect this may account for the TNT-like fragments we observed in the fixed sample noted earlier (Fig. 3A, yellow box/arrows). These results strongly suggest that MARCKS ED influences heterotypic TNTs.

### PKC stimulation increases TNTs between NHAs and JX14

**Fig. 4:**
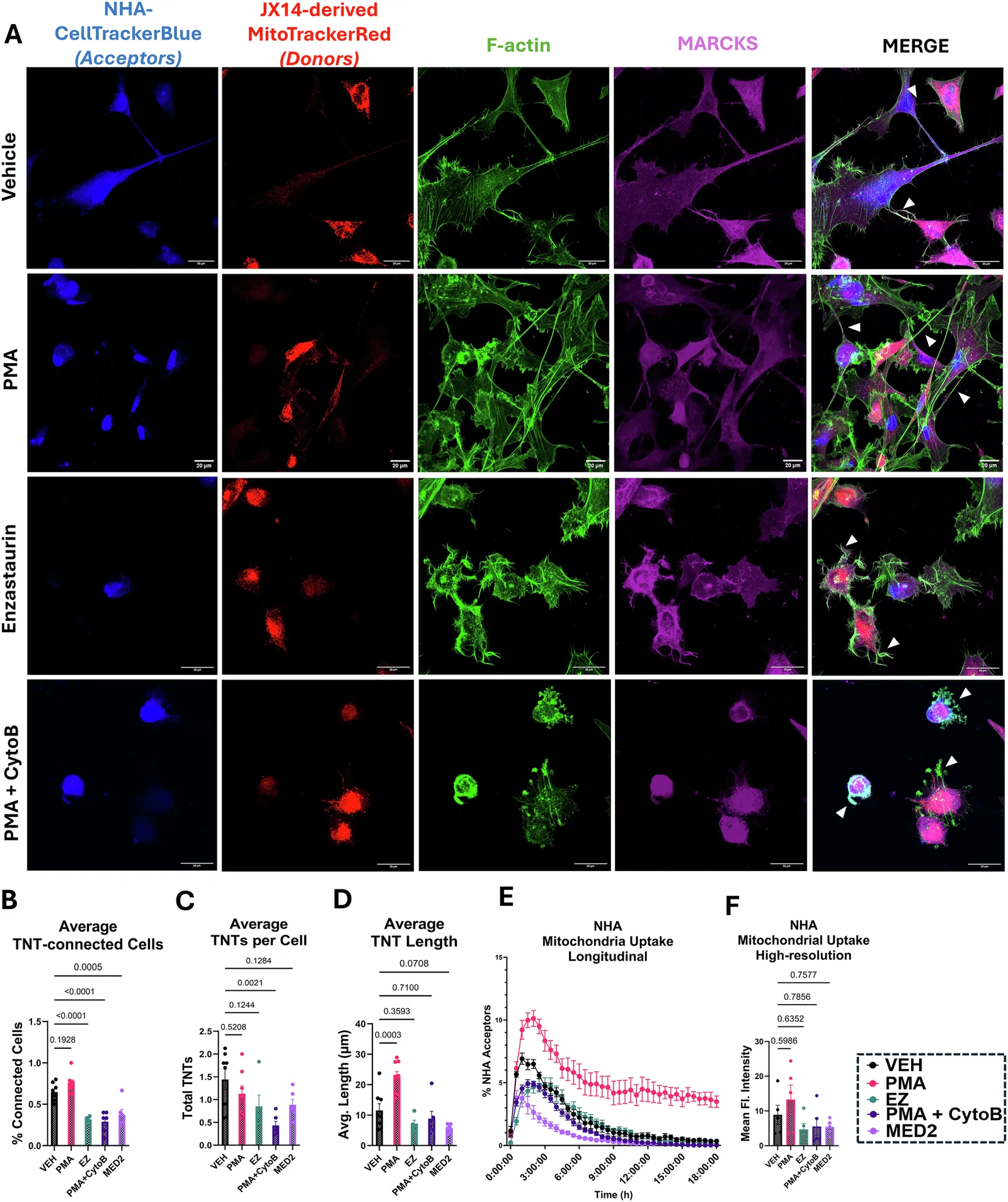
PKC stimulation increases TNTs between JX14 and NHAs. **A** Representative maximum-intensity widefield fluorescent images of NHAs and JX14 directly co-cultured under different treatment conditions for 4 h (1 µM PMA, 3 µM EZ, and 1 µM PMA + 1 µM CytoB), 20 µm scale bar. White arrows indicate TNT-like structures. **B** Average number of cells connected by TNTs ****p < 0.0001 (VEH vs EZ, actual *p* < 0.0001; VEH vs PMA +CytoB, actual *p* < 0.0001) ***p < 0.001 (VEH vs MED2, actual *p* = 0.0005) by one-way ANOVA with Dunnett’s comparison. **C** Average TNTs quantified per cell **p < 0.01 (VEH vs PMA +CytoB, actual *p* = 0.0021) by one-way ANOVA with Dunnett’s comparison. **D** Average length of TNTs normalized to total cell number ***p < 0.001 (VEH vs PMA, actual *p* = 0.0003) by one-way ANOVA with Dunnett’s comparison. **E** Percentage of NHAs with JX14-derived mitochondria quantified longitudinally every 30 min over 18 h **p < 0.01 (2 h Peak, VEH vs PMA, actual *p* = 0.0090, VEH vs MED2, actual *p* = 0.0027), *p < 0.05 (VEH vs PMA+CytoB, actual *p* = 0.0449) by two-way ANOVA with repeated measures and Šidák’s comparison. **F** Mean fluorescent intensity of NHA mitochondria confocal images at 3.5 h. TNT metrics, *N* = 8 VEH, *N* = 8 PMA, *N* = 5 EZ, *N* = 6 PMA + CytoB, *N* = 6 MED2 were analyzed per experimental condition out of 3 independent cultures. Mitochondrial transfer, *N* = 5 were analyzed per experimental condition out of 3 independent cultures. All graph error bars represent SEM.

Since MARCKS phosphorylation is largely regulated by PKC, we investigated the effect of PKC stimulation on TNTs. Cells were treated with respective PKC-targeting drugs for a total of 3.5 h before fixation. Phorbol 12-myristate 13-acetate (PMA) was utilized to stimulate PKC, while Enzastaurin (EZ) inhibited PKC. We observed increased heterotypic TNT-like structures positive for anti-MARCKS between JX14 and NHAs under vehicle conditions and upon induction of PKC activation with PMA, which promotes MARCKS phosphorylation (Fig. 4A–D). In contrast, we observed abnormal directionality of TNT-like structures when treated with EZ, resulting in decreased TNT-connected cells, TNTs per cell, and average TNT length compared to the control (Fig. 4A–D). Interestingly, the TNT phenotype induced by PMA treatment was partially restored upon co-administration with the TNT inhibitor Cytochalasin B (CB) (Fig. 4A–D). Kinetic measurements of mitochondrial uptake by NHAs over 18 h were obtained every 30 min, during which we observed the peak of transfer around 2 h. PMA-treated cells exhibited a significant increase in mitochondria uptake compared to all other conditions (Fig. 4E). MED2 resulted in the least amount of mitochondria uptake, followed closely by EZ and PMA + CB (Fig. 4E). We also quantified mitochondrial intensity in NHAs at 3.5 h, showing similar trends (Fig. 4F). These results support PKC activity to be an influential element in TNT formation. JX14 was also assessed in monoculture under these conditions; however, JX14 exhibited less homotypic TNT-related alterations, suggesting the potential primary utilization of TMs over TNTs (Supplementary Fig. S1).

### U87 MARCKS effector domain mutants have altered TNT phenotypes

**Fig. 5:**
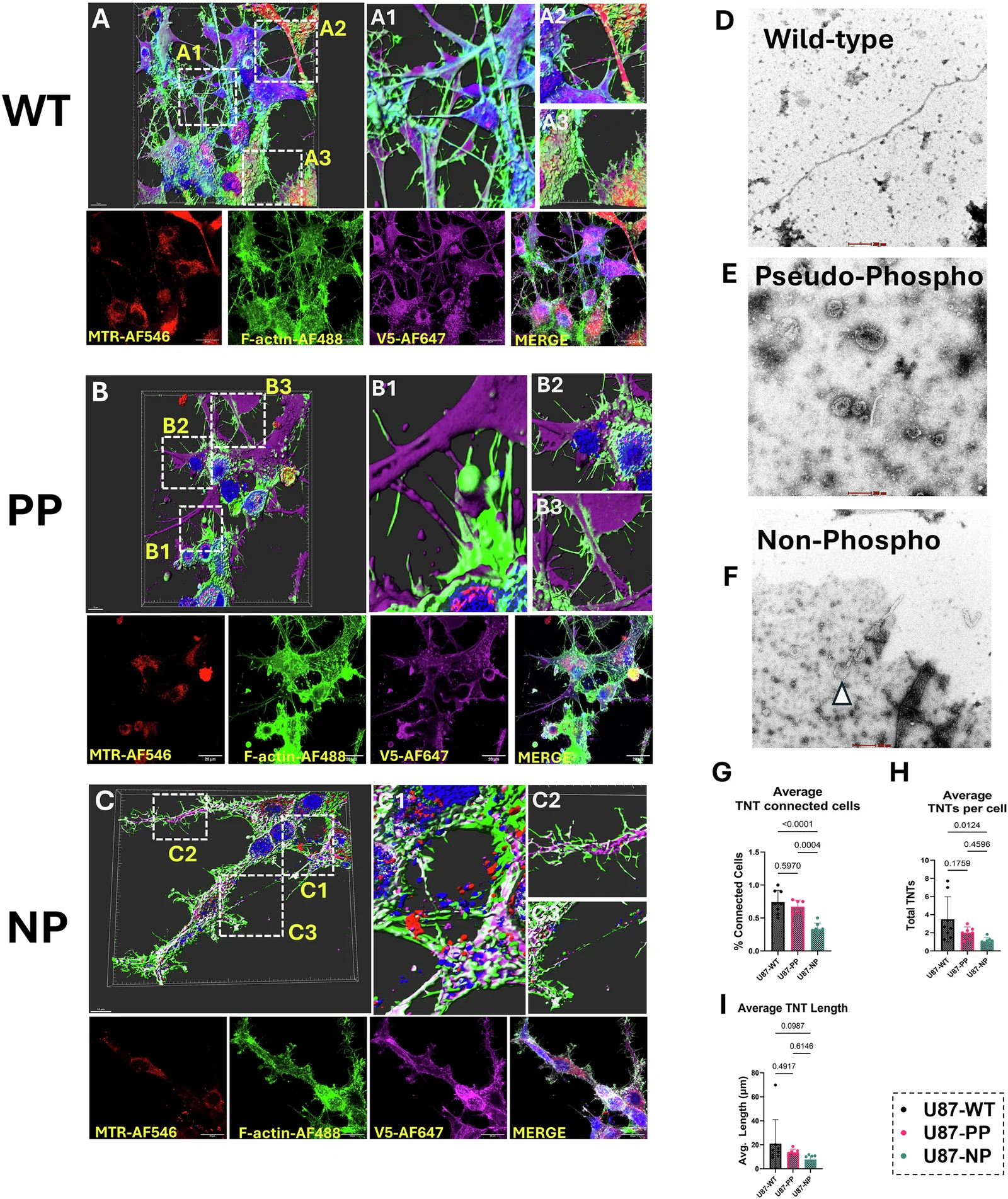
U87 MARCKS ED mutants exhibit differential TNT morphology. Representative, Imaris-rendered, maximum-intensity widefield fluorescent images of U87 MARCKS ED mutants following 72 h doxycycline-induction, indicated by V5-positive cells. **A** U87-WT depicting several views of homotypic TNTs (A1) indicated with a 10 µm scale bar or in magnified ROI’s depicting numerous views of TNTs in U87-WT (A1-3). Bottom panel shows individual fluorescent channels with indicated antibody targets, 20 µm scale bar. **B** U87-PP showing different views of TNTs (B1) indicated with a 10 µm scale bar or as magnified ROI’s showing U87-PP’s ability to form TNTs (B1-3). Bottom panel shows individual fluorophore channels with antibody targets indicated, 20 µm scale bar. **C** U87-NP depicting several views of TNTs as a whole (C1) indicated with a 10 µm scale bar or in magnified ROI’s (C1-3). Bottom panel shows individual fluorophore channels with indicated antibody targets, 20 µm scale bar. Negative stain TEM of U87-WT (**D**), U87-PP (**E**), and U87-NP (**F**) indicated with a 200 nm scale bar. U87-NP exhibits clubbed tips (white arrow) while filaments maintain integrity in U87-WT and U87-PP. **G** Average TNT-connected cells ****p < 0.0001 (WT vs NP, actual *p* < 0.0001, WT vs PP, actual *p* = 0.0004) by one-way ANOVA with Tukey’s comparison. **I** Average TNTs per cell **p < 0.01 (WT vs NP: *P* = 0.0089) by one-way ANOVA with Tukey’s comparison. **H** Average TNT length. *N* = 8 U87-WT, *N* = 6 U87-PP, *N* = 8 U87-NP were analyzed per experimental condition out of three independent cultures. All graph error bars represent SEM.

To further investigate MARCKS ED function in TNT formation in JX14 and NHAs, we utilized U87 cells overexpressing V5-tagged MARCKS with ED mutations, including wild-type (WT), non-phosphorylated (NP), or pseudo-phosphorylated (PP), cultured in PDX (serum-free) conditions to promote TNT dynamics. We first sought to characterize homotypic TNTs in the modified MARCKS ED U87s (full validation of doxycycline-inducible mutations detailed previously) [28, 30, 48]. Anti-V5 was probed to ensure sufficient mutation induction and visualization of mutated MARCKS localization. U87-WT cells exhibited a high abundance of TNT–like structures, forming extensive intercellular networks that connected the majority of cells within the field of view (Fig. 5A). Upon induction of U87-PP, numerous TNT-like structures were observed that maintained comparable morphology and structural integrity, closely phenocopying the conditions observed following PMA treatment (Fig. 5B). In contrast, U87-NP cells displayed pronounced cellular aggregation characterized by membrane adhesion, accompanied by a marked reduction in TNT-like structures, which also exhibited impaired directionality and decreased structural stability (Fig. 5C). Next, we used negative stain transmission electron microscopy (TEM) on each cell line to investigate TNT morphology. U87-WT exhibited long, branched TNT-like filaments, while U87-PP filaments appeared shorter, though still maintaining integrity (Fig. 5D, E). Strikingly, U87-NP exhibited TNT-like filaments with clubbed tips (white arrow) or deformed structure (Fig. 5F, white arrow). In line with these results, we quantified fewer average TNTs per cell, TNT-connected cells, and TNT length in U87-NP compared to both U87-PP and U87-WT (Fig. 5G–I). These results phenocopy the morphological and functional aspects observed with PKC activation and inhibition, supporting a potential role for MARCKS ED in TNT dynamics.

### NHA co-cultures with U87 MARCKS mutants phenocopy pharmacological findings

**Fig. 6:**
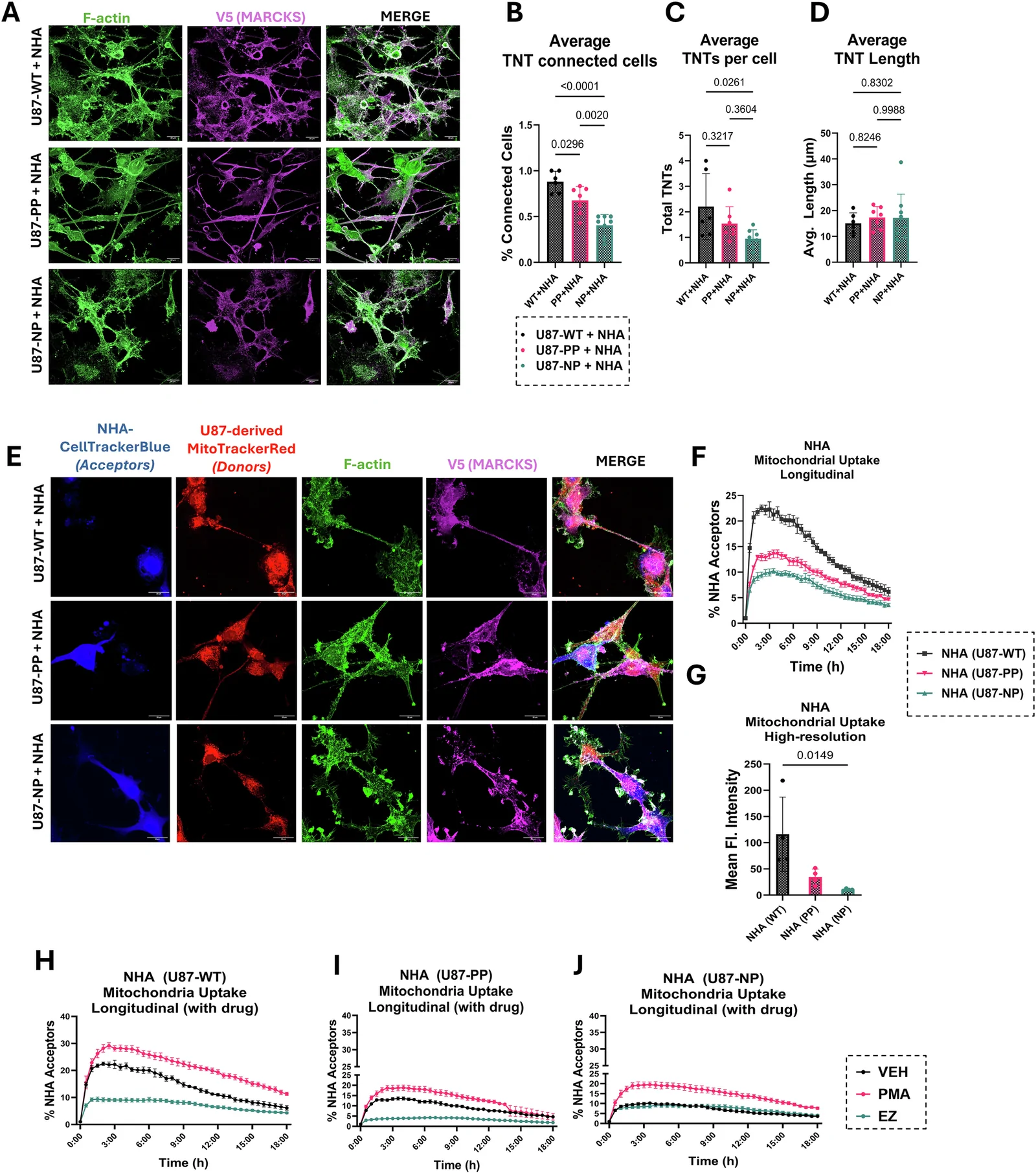
NHA co-cultures with U87 MARCKS mutants closely mimic pharmacologically stimulated phospho-events with JX14. **A** Representative maximum-intensity widefield fluorescent images of induced U87-WT, -NP, or –PP cocultured with NHAs, 20 µm scale bar. **B** Average TNT-connected cells across conditions ****p < 0.0001 (WT vs NP, actual *p* < 0.0001) **p < 0.01 (PP vs NP, actual *p* = 0.0020), *p < 0.05 (WT vs PP, actual *p* = 0.0296) by one-way ANOVA with Tukey’s comparison. **C** Average number of TNTs per cell *p < 0.05 (WT vs NP, actual *p* = 0.0261) by one-way ANOVA with Tukey’s comparison. **D** Average TNT length. **E** Representative maximum-intensity widefield fluorescent images of U87-derived mitochondrial transfer to NHAs. **F** Percentage of NHAs with U87-derived mitochondria quantified longitudinally every 30 min over 18 h ****p < 0.0001 (2 h Peak, NHA-WT vs NHA-NP, actual *p* < 0.0001), ***p < 0.001 (2 h Peak, NHA-WT vs NHA-PP, actual *p* = 0.0002), *p < 0.05 (2 h Peak, NHA-PP vs NHA-NP, actual *p* = 0.0218) by two-way ANOVA with repeated measures and Tukey’s comparison. **G** Mean fluorescent intensity of NHA confocal imaging at 3.5 h *p < 0.05 (NHA-WT vs NHA-NP, actual *p* = 0.0149) by one-way ANOVA with Tukey’s comparison. **H** Percentage of NHAs with U87-WT derived mitochondria ****p < 0.0001 (2 h peak, VEH vs EZ, actual *p* = 0.0001), ***p < 0.001 (2 h peak, PMA vs EZ, actual *p* = 0.0003), *p < 0.05 (2 h peak, VEH vs PMA, actual *p* = 0.0424) by two-way ANOVA with Tukey’s comparison. **I** Percentage of NHAs with U87-PP derived **p < 0.01 (VEH vs EZ, actual *p* = 0.0011, PMA vs EZ, actual *p* = 0.0028) by two-way ANOVA with Tukey’s comparison. **J** Percentage of NHAs with U87-NP derived mitochondria **p < 0.01 (PMA vs EZ, actual *p* = 0.0063), *p < 0.05 (VEH vs PMA, actual *p* = 0.0120) by two-way ANOVA with Tukey’s comparison. TNT metrics, *N* = 8 U87-WT, *N* = 6 U87-PP, *N* = 8 U87-NP were analyzed per experimental condition out of 3 independent cultures. Mitochondrial transfer, *N* = 4 were analyzed per experimental condition out of three independent cultures. All graph error bars represent SEM.

Next, we aimed to characterize heterotypic TNT dynamics between U87 MARCKS ED mutants co-cultured with NHAs. Co-cultures were maintained in the same conditions as JX14 and NHAs. We noted increased TNT-like structures and TNT-connected cells in the -WT and –PP, but not in the –NP co-culture group (Fig. 6A–D). Interestingly, average TNT length was not significantly different in all groups, suggesting astrocytes may partially rescue this phenotype in –NP groups (Fig. 6D). Next, TNT functionality was assessed by examining mitochondrial transfer from U87 cells to NHAs. NHAs received increased mitochondria from U87-WT cells, followed by U87-PP, and least from U87-NP, possibly indicating endogenous MARCKS activity contributes to increased transfer (Fig. 6E, F). We then stimulated PKC in all co-cultures to detect any further changes in mitochondrial dynamics. PMA-treatment increased mitochondrial transfer to NHAs compared to vehicle and EZ-treatment conditions (Fig. 6G–I). This suggests PKC activity may promote MARCKS phosphorylation in NHAs, increasing NHA-derived TNTs, which may also function to uptake mitochondria.

### MARCKS co-expression in the public database

**Fig. 7:**
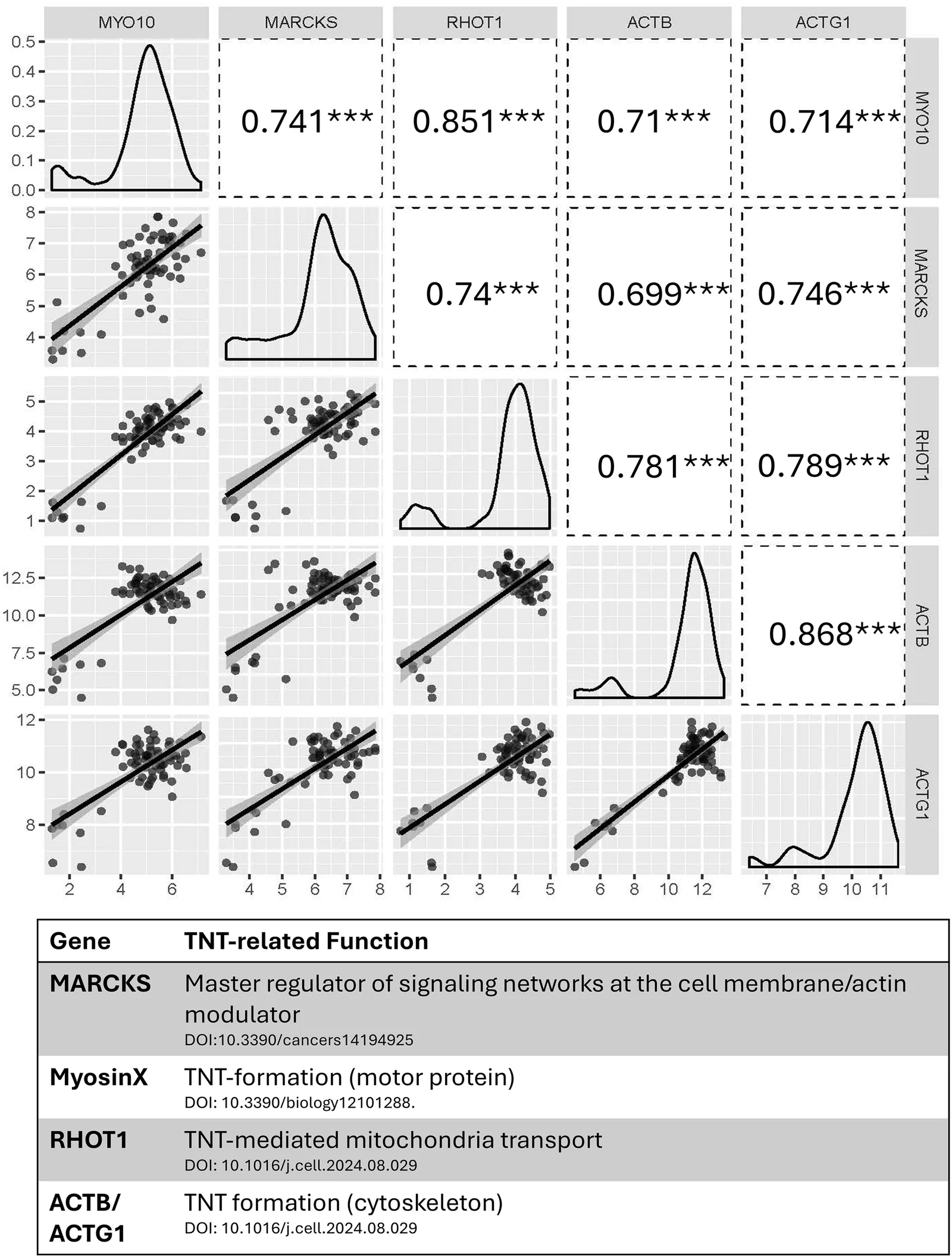
MARCKS positively correlates with TNT-related protein-encoding genes in primary classical GBM. Pearson’s Correlation between various proteins. Obtained from CGGA GlioVis public patient data with parameters set to primary classical GBM. ****p < 0.0001 (MARCKS vs. MyosinX, R^2^ = 0.741, MARCKS vs. ACTB, R^2^ = 0.699, MARCKS vs. ACTG1, R^2^ = 0.746, MARCKS vs. RHOT1, R^2^ = 0.740), actual *p* < 0.0001.

To further support MARCKS’ regulation of TNTs in GBM, we investigated public patient data available on GlioVis using the Chinese Glioma Genome Atlas (CGGA) dataset [50]. For relevance purposes, we set the parameters to include only CGGA data for primary classical GBM since JX14 is a primary, classically derived GBM xenoline [42, 51]. We determined the correlation between MARCKS and other genes that are implicated in TNT formation and TNT-mediated mitochondrial trafficking: MyosinX (motor), ACTB and ACTG1 (actin-binding), and RHOT1 (mitochondria trafficking) [19, 23, 52, 53]. MARCKS exhibited strong positive correlations with all these protein-encoding genes, suggesting some potentially overlapping signaling dynamics that could aid in TNT regulation (MARCKS vs. MyosinX (R^2^ = 0.741); vs. ACTB (R^2^ = 0.699); vs. ACTG1 (R^2^ = 0.746); vs. RHOT1 (R^2^ = 0.740); *P* < 0.0001) (Fig. 7).

## Discussion

The current study provides evidence supporting an important role for MARCKS in heterotypic TNT regulation between GBM cells and NHAs. PKC stimulation, well-known to phosphorylate MARCKS ED, appears to be influential on proper TNT formation and function. Lentivirus overexpression of wild-type and pseudo-phosphorylated MARCKS in U87s further supported these findings, as both conditions enhanced TNT formation and function compared to the non-phosphorylatable MARCKS ED mutant. In addition, we show strong positive correlations between MARCKS and TNT-related protein-encoding genes in classical, primary GBM patient data, obtained from the Chinese Glioma Genome Atlas (CGGA) using GlioVis [50, 51].

PKC and downstream regulation of TNTs have been minimally investigated previously and are not well explored in the context of the brain or GBM, especially in relation to MARCKS [46]. Importantly, we demonstrate MARCKS’ localization in TNTs, and both PKC manipulation and MARCKS ED phospho-mutant expression alter TNT phenotypes. Interestingly, we were able to rescue increases in PMA-treated TNTs with simultaneous treatment of the TNT-inhibitor, Cytochalasin B (Fig. 4B–F). Collectively, we observed increased TNT-connected cells and mitochondrial transfer to NHAs when stimulating PKC with PMA, and overexpressing WT or pseudo-phosphorylated MARCKS (Figs. 4B and 6B).

Using fixed and live cell confocal imaging, we were able to determine that TNTs form readily between our stem-like GBM model, JX14, and NHAs; however, the timing of this process is significantly delayed when treated with MED2 (Fig. 3G, H, Supplementary Video SV2 and SV3). MED2-treated TNTs presented with altered morphology and functionality, further supporting the role of MARCKS ED in TNT dynamics. Decreased TNT dynamics following EZ treatment or simultaneous PMA and Cytochalasin B treatment were also observed.

There are several limitations in the present study. The usage of pharmacological agents over genetic or lentivirus modification in our most physiologically relevant GBM model, JX14, can be perceived as a weakness. However, we were able to support our findings using U87-MARCKS ED mutants that possess the same null PTEN status as the JX14 model, making both models suitable for MARCKS exploration studies. Additionally, by using PKC-targeting drugs, we were able to discern a role for PKC upstream of MARCKS. Another drawback is the current state of the TNT field, which lacks a single biomarker for TNTs, resulting in TNT assessments being largely based on three-dimensional (3D) morphology, proper staining, and organelle-passaging abilities [54, 55]. We attempted to circumvent these challenges by demonstrating 3D live and fixed confocal imaging, showing TNTs floating above the substratum and containing mitochondria (Figs. 2D; 3G, SV1, SV2, and SV3). Much debate still exists regarding the semantics of membrane-derived tubes. “Thin” and “thick” TNTs versus TMs have reported similarities and differences, further complicating the discovery of a single biomarker. Recent studies in B-Lymphoma cells have indicated that “thick” TNTs contain microtubules, which raises debate over what constitutes a TNT compared to a TM, or if they are transient products of each other [56]. Nonetheless, the current state of the field considers them to be distinct entities, and because TNTs are prevalent in several diseases, proper terminology could be cell-type or disease-specific [57]. Branching off this, it is important to acknowledge that our co-culture system does not represent the complexity of the heterogeneous TME; therefore, TNT-related signaling pathways may differ amongst various non-tumor cell populations when in contact with tumor cells. Though limiting, it was necessary to focus only on tumor cell outcome in the current study. Therefore, it will be important to determine the reciprocal effect on NHA-derived mito-transfer and NHAs that have received GBM-derived mitochondria from cells with altered MARCKS phosphorylation in the future. Additional tumor models can also be examined to determine whether variations in MARCKS levels and its endogenous phosphorylation influence TNTs. Indeed, prior studies have identified MARCKS as a potential gene biomarker for GBM “stemness” using-single cell cultures from GBM patients [58]. Due to clonal evolution of stem-like brain tumor-initiating cells, there is a possibility that clinical therapies (e.g., radiation and temozolomide) will lead to clonal selection such that the originally active TNT-related pathway may compensate by utilizing alternative pathways during the selection process [59]. Future studies using our acquired therapeutic resistance models could help identify these potential alterations [60, 61].

Despite these limitations, we anticipate that MARCKS-related therapies, such as MED2, could be successfully combined with other cytotoxic therapies for GBM treatment in the future. In the current study, JX14 exposed to NHAs had increased viability following TMZ treatment compared to monoculture (Fig. 1). We are eager to investigate if this survival advantage is diminished upon simultaneous treatment with MED2. We have previously shown MED2 is not cytotoxic to NHAs unless a considerably high concentration is administered; however, GBM xenolines are sensitive at low to moderate doses [42]. We postulate that coupling MED2 with therapies that are suggested to spare normal cells (i.e., tumor treating fields, FLASH radiation) will result in increased normal cell protection and tumoral cytotoxicity due to the reduction in TNT-mediated cellular interactions between GBM cells and NHAs [62, 63].

Importantly, these findings hold strong translational relevance for emerging efforts to therapeutically target MARCKS’ signaling. A growing body of translational and clinical literature highlights MARCKS, particularly its phosphorylation status, as a druggable target downstream of PKC across cancer, with multiple MARCKS targeting peptides (e.g., MANS, BIO11006, MPS, and MED2) advancing through preclinical and early phase clinical development [64]. Existing translational and clinical studies have largely focused on MARCKS N-terminal myristoylation–targeting peptides (e.g., MANS and its derivative BIO 11006), which disrupt membrane association and downstream signaling [65]. Furthermore, they have shown favorable toxicity profiles in early-phase clinical trials for inflammatory diseases and cancer [64, 65]. In contrast, ED-targeting peptides (e.g., MPS or ED mimetics like MED2) directly modulate MARCKS phosphorylation dynamics and intracellular effector functions [64]. In preclinical cancer models, MPS or ED mimetics appear more effective at suppressing tumor-specific behaviors such as invasion, stemness, and treatment resistance [64, 66]. Our data builds on this work by identifying a previously unrecognized role for MARCKS ED phosphorylation in regulating TNT formation and mitochondrial transfer between GBM cells and NHAs, both being processes implicated in tumor microenvironment crosstalk and treatment resistance. Notably, several MARCKS-directed peptides selectively modulate MARCKS phosphorylation without inducing overt toxicity in normal cells [64], suggesting that TNT attenuation via MARCKS targeting may be therapeutically feasible while sparing healthy brain tissue. The correlation of MARCKS with TNT-related protein-encoding genes in primary GBM patient datasets further strengthens clinical relevance and underscores its potential as a biomarker or therapeutic target.

Our findings suggest that MARCKS ED phosphorylation and PKC activation promote TNT formation and influence TNT functionality (Supplementary Fig. S2). We believe the field of cellular communication and GBM will greatly benefit from these insights, and other groups will be able to extend our findings in the future. Importantly, elucidating the role of MARCKS ED and upstream PKC in GBM-NHA TNT regulation may reveal a new potential target or combinatorial therapy for overcoming treatment resistance in GBM. To the best of our knowledge, this is the first study investigating the role of MARCKS’ ED in TNT regulation between NHAs and GBM cells (i.e., BTICs or U87s). We anticipate our work will contribute to advancing the existing TNT and cellular communication field in GBM and other brain-related diseases.

## Materials and methods

### Cell culture conditions

#### Patient-derived xenograft (PDX) media

Dulbecco’s Modified Eagle Medium (DMEM)/F12 50/50 (Corning, Cat. #10-090-CV), B27 supplement (50X) Gibco, Cat.#17504044, 1% penicillin–streptomycin (Corning, Cat.#30-001-CI), 1% sodium pyruvate (Corning Cat.#5000CI), EGF (20 ng/mL), FGF (20 ng/mL).

#### Serum-free astrocyte (SFA) media

Neurobasal medium (Gibco, Cat. #12349015) and Dulbecco’s Modified Eagle Medium (DMEM)/F12, 50/50 (Corning, Cat. #10-090-CV) (1:1 v/v) supplemented with 1% Penicillin–streptomycin (Corning, Cat.#30-001-CI), 1 mM sodium pyruvate (Corning, Cat.#5000CI), bovine serum albumin (100 mg/L) (Sigma Cat.# A9418-5G), N2 supplement (100X) (Gibco, Cat.#17502048), N-Acetyl Cysteine (2.5 mg/L) (Thermofisher, Cat. #160280250), Human EGF-basic (20 ng/mL) (Gibco, Cat. #PHG0311), and Human FGF (20 ng/mL) (Gibco, Cat. #PHG0261). Serum-free astrocyte model validated as previously described [47].

#### Cell lines

JX14 (also known as “GBM14” or “JX14P”) was purchased from Mayo Clinic and maintained in the UAB Brain Tumor Model Core. JX14 was kept in PDX media and re-passaged in the flank of athymic nude mice after passage 10 in vitro. PDX Generation described previously [67]. Their use was approved by the UAB IRB (IRB-300002910). Normal human astrocytes (NHA) were purchased from Lonza (cat. #CC-2565) and kept in SFA media. Experiments were performed in accordance with UAB guidelines and regulations.

U87 MARCKS mutants were established as previously described [30, 45]. In long-term culture, U87 cells were maintained in a 1:1 mixture of PDX media and DMEM/F12 50/50 (Corning, Cat. #10-090-CV) supplemented with 5% fetal bovine serum (FBS) (Sigma, Cat. #F0926). U87 cells were placed in complete PDX media 3 days prior to induction. 300,000 cells were plated in a 60 mm petri dish and induced with 2 µg/ml of doxycycline in PDX media for 72 h. For experiments, cells were maintained in only PDX media +/– doxycycline to promote stem-like properties and TNT formation. U87 MARCKS mutant and normal human astrocyte co-cultures were maintained in a 1:1 medium of PDX +/– doxycycline and SFA for experimental purposes. STR validation and mycoplasma testing were performed on cell lines.

### Drug experiments

Drug concentrations and vendor sourcing are listed in Table 1 below. GBM PDX and NHA cells were treated with TMZ for 5 days before cytotoxicity analysis. GBM PDX or U87 cells and NHAs were treated in co-culture with the listed concentrations of PMA, EZ, MED2, and Cytochalasin B for three h and 30 min before fixation and analysis.

**Table 1:** Drug experiment concentrations and vendor sourcing information.

Drug	Concentration	Vendor
Temozolomide (TMZ)	150 µM	Sandoz, Basel, Switzerland, cat. # 0781-2694-44
Phorbol-Myristate Acetate (PMA)	1 µM	Sigma Aldrich, Cat #P8139
Enzastaurin (EZ)	3 µM	Selleckchem, Cat. #S1055
MARCKS Effector Domain Peptide (MED2)	3 µM	Established previously [42]
CytochalasinB	1 µM	Sigma Aldrich, Cat # 250233

### Immunocytochemistry (Nikon A1R-HD25 Ti2)

200,000 cells were seeded in monoculture or in co-culture at a 1:1 ratio onto Geltrex-coated (Gibco, Cat. #A1413302) 12 mm coverslips (Neuvitro, Cat. #NC0706236). Cells were fixed with a 1:1 ratio of 4% PFA and 0.05% glutaraldehyde for 15 min. Cells were quenched for 10 min with 100 mM NH4Cl and then permeabilized with 0.1% Triton-X for 3 min.

*Protocol adapted from: Inés Sáenz-De-Santa-María, J Michael Henderson, Anna Pepe, Chiara Zurzolo. Identification and Characterization of Tunneling Nanotubes for Intercellular Trafficking. Current Protocols, 2023, 3* [11]*, pp.e939. ff10.1002/cpz1.939ff. ffpasteur-04315571f*

Cells were stained with anti-MARCKS (Abcam, Cat. #1446Y), anti-TNFAIP2 (Invitrogen, Cat. #PA5-34708 or sc-28318), anti-V5 (Invitrogen, Cat. #A01805-100), Phalloidin (Invitrogen, Cat. # A12379 or Cat. #R415), according to the manufacturer's protocol. Cells were incubated with secondary antibodies conjugated to AF-488 (Thermofisher, Cat # A32731) or AF-647 (Thermofisher, Cat # A32733), respectively. Images were obtained on a Nikon A1 Confocal.

### Indirect co-culture viability assay

500,000 GBM cells (JX14 or U87) were seeded onto Geltrex-coated (Gibco, Cat. #A1413302) 6 well plates. A 1.0 µm Transparent PET Membrane (Corning, Cat. #353102) was placed in the well, and 500,000 NHAs were seeded onto the insert. Samples were then incubated for 5 days, and then individually assessed for viability using CellTiter-Glo 2.0 (Promega Cat: G9241).

*Protocol adapted from* [68]*: Thayanithy, V., O’Hare, P., Wong, P. et al. A transwell assay that excludes exosomes for assessment of tunneling nanotube-mediated intercellular communication. Cell Commun Signal 15, 46 (2017)*. https://doi.org/10.1186/s12964-017-0201-2 [69].

### Mitochondria transfer assay

#### Direct contact, cytation5

5000 NHAs were stained with 5 µg/ml cell tracker blue according to the manufacturer's protocol and designated “Acceptor cells”. 5000 JX14 or induced U87 cells were separately stained with 500 nM MitoTrackerRed according to the manufacturer's protocol and were designated “Donor cells”. All groups were plated in a black-walled 96-well plate and were imaged immediately after plating on the Cytation5 every 30 min for 18 h. Double positive (acceptor cells containing mito-tracker) were quantified automatically using the Gen5 software. Values were normalized by dividing the later timepoints by time 0 h. Values represent quadruplicates repeated three separate times. Statistical analysis was conducted in GraphPad Prism as a two-way ANOVA with repeated measures and multiple comparisons.

#### Direct contact, Nikon A1R

200,000 GBM cells (JX14 or U87) and NHAs per well were seeded at a 1:1 ratio and stained as described above. Cells were fixed as described in the immunocytochemistry section. Z-stacked images were acquired with a Nikon A1R confocal microscope. MitoTracker Red accumulation in at least 40 acceptor cells (NHA) was quantified automatically using machine learning from three biological replicates in Bitplane Imaris 10.1. Further details are provided in Appendix 1 of the supporting information.

### TNT characterization

200,000 cells were seeded in monoculture or in co-culture at a 1:1 ratio as described above. Cells were stained with the appropriate markers described above. Homotypic and heterotypic TNTs and TNT-connected cells were identified in images with a blinded sample number along the Z-plane using ICY Bioimage software (version 2.4.3.0) TNT manual plug-in https://icy.bioimageanalysis.org/. Average length was quantified along the Z-plane using FIJI (version 2.14.0). At least 100 cells were assessed per condition from three biological replicates. Further details are provided in Appendix 1 of the supporting information.

### Cytation5 cell imaging multimode reader

Samples were imaged on an Agilent Cytation5 fluorescence widefield microscope, equipped with a xenon flash lamp (Agilent), using an Olympus 4x/0.13 NA air lens, a 4x Phase/0.1 NA air lens, 20x/20x Phase 0.45 NA air lens. EGFP was detected using a 469/35 nm EX (Agilent), 525/39 nm EM (Agilent), and 497 nm dichroic mirror (Agilent). Texas Red was detected using a 586/15 nm EX (Agilent), 647/57 nm EM (Agilent), and 605 nm dichroic mirror. The samples were acquired with a Sony IMX 264 CMOS WFOV camera (1992 × 1992 pixels), pixel size: 3.45μm. Gen5 software (v3.15) was employed to quantify fluorescently stained cells.

### Confocal microscopy image acquisition

Samples were acquired on a Nikon Ti2 inverted fluorescence microscope with a tandem galvano and Nikon A1R-HD25 resonance scanner. Images were acquired with 1024×1024 or 2048×2048 Nyquist pixel dimensions using an Apo 60x/1.4 NA gamma oil DIC wd 140 objective. EGFP was detected using a 488 nm laser, TexasRed was detected using a 567 nm laser, DAPI was detected using a 405 nm laser, and far red (Cy5) was detected using a 637 nm laser. NIS Elements 5.0 imaging software was used to acquire Z-stack images (55-60 slices per image).

### Live cell confocal (Nikon A1R-HD25 Ti2)

NHAs were stained with 1 µM CellMask™ Orange Actin Tracking stain based on the manufacturer’s protocol (Thermofisher Cat. #A57244). 200,000 cells were seeded at a 1:1 concentration (100,000 of each cell type) onto geltrex-coated 35 mm coverglass petri dishes (Mattek Cat. # P35G-1.5-14-C) and allowed to settle for 2 h. Cells were imaged at 60x/1.4NA on Nikon A1R-HD25 Ti2 eclipse inverted microscope. During imaging, cells were housed in a live cell temperature (37 °C) and gas-controlled (5% CO2) Tokai Hit incubation stage chamber to mimic incubator conditions. Nis Elements 5.0 imaging software was used to acquire Z-stack images at 1024×1024 pixels every 3 min for 1.5 h. Timelapses were then 3D-rendered in Imaris or NIS Nikon Elements.

### Negative stain transmission electron microscopy (TEM)

Samples were induced for 72 h in PDX media, then collected to assess morphological changes present in mutant TNTs with negative stain TEM. 300 mesh carbon film, copper EM grids (EMS) were glow-discharged using a PELCO easiGlow 9000 system (Ted Pella). 4.5 mL of sample was applied to each grid and incubated for 1 min. Excess liquid was blotted away on blotting paper. One drop of 2% uranyl acetate (UA) solution (EMS) was applied to the grid, followed by blotting off excess. This step was repeated three times in total. The grids were then allowed to dry for 5 min prior to storage. Negative stain TEM images were collected on a 120 kV Technai T12 transmission electron microscope (TEM) (FEI) with a 4k CCD camera and total dose of 20-30 e-/Å2.

### Statistical analysis

All analyses were conducted with Prism v10.1 (GraphPad Software). Data are presented as mean ± SEM. For all analyses, the experimenter was blinded to the sample number until it was time to graph the results. Identification of TNTs and TNT-connected cells was manually done as described above and in Appendix 1 of the Supporting Information. For quantification of TNTs, TNT-connected cells, and average TNT length, at least a total of 100 cells per condition were assessed from three separate biological replicates. Average TNT-connected cells were generated by dividing the total number of TNT-connected cells by the total number of cells present. The average TNTs per cell was calculated by dividing the total TNTs by the total number of cells present. Lengths of TNTs were measured based on membrane fluorescence, from one end of the protrusion to the end of the adjacent cell, then averaged per replicate. Statistical tests, comparisons, and sample numbers are indicated in each figure legend.

## Supplementary information

Supplemental Figure Legends

Supporting Information Appendices

Supplemental Figure S1

Supplemental Figure S2

Supplemental Video SV1

Supplemental Video SV2

Supplemental Video SV3

## Data Availability

All data related to the manuscript are available in the main or supplementary figures. Data can be made available upon request to the corresponding author. The following table belongs within the methods of the main manuscript underneath the text below the headline “Drug Experiments”.
