# Supplementary material for: MARCKS as a mediator and target for pathological tunneling nanotubes between patient-derived glioblastoma cells and astrocytes
Source: Cell Death Dis. 2026 Jul 13;17(1):867. doi: 10.1038/s41419-026-09109-x (PMC13642262; doi:10.1038/s41419-026-09109-x)
Supplement: Supplementary file 1 [file 41419_2026_9109_MOESM1_ESM.docx]

**Supplemental Figure S1: Homotypic TNT Dynamics in JX14. A**) Representative maximum-intensity widefield fluorescent images of JX14 cells under different PKC-targeting drug conditions. **B**) Average TNT-connected cells **p<0.01 (VEH vs PMA+CytoB, actual p=0.0031), *p<0.05 (VEH vs EZ, actual p=0.0293) by one-way ANOVA with Dunnett’s comparison. **C**) Average TNTs per cell. **D**) Average TNT length **p<0.01 (VEH vs PMA +CytoB, actual p=0.0072), *p<0.05 (VEH vs PMA, actual p=0.0285 by one-way ANOVA with Dunnett’s comparison. E) Percentage of JX14 with JX14-derived mitochondria quantified longitudinally every 30min over 18h ****p<0.0001 (2h Peak, VEH vs PMA, actual p<0.0001, VEH vs EZ, actual p<0.0001, VEH vs PMA+CytoB, actual p<0.0001) by two-way ANOVA with repeated measures and Šidák’s comparison. N = 4 VEH, N = 3 PMA, N = 3 EZ, N = 3 PMA + CytoB were analyzed per experimental condition.

**Supplemental Figure S2:** **Proposed MARCKS-related TNT mechanism**. MARCKS is typically bound to the PIP_2_ at the cell membrane. Upon phosphorylation by PKC, MARCKS is released from PIP_2_ and TNT protrusions result from the dissociation. MARCKS may also be crosslinking necessary actin at the cell membrane in preparation for TNT protrusions. Created in BioRender. Nassour, L. (2026) https://BioRender.com/9uvzae8

**Supplemental Video SV1: Three-Dimensional View of TNT Suspended Above the Substratum Between NHA and JX14 Under Control Conditions.** High-resolution confocal image processed in Imaris to show maximum intensity projection and 3D orientation of TNT. Video shows JX14 (MitoTrackerRed-AF546) and NHA (CellTrackerBlue) connected by TNT positive for F-actin (AF488) containing MARCKS (anti-MARCKS-AF647), and JX14-derived mitochondria.

**Supplemental Video SV2: Live Cell Confocal Time-lapse Depicting Transient TNT Formation, Extension, and Retraction between NHA and JX14.** High-resolution confocal image frames captured every three minutes using NIS Elements. Sequential images were processed in Imaris to show maximum intensity projection of TNT. Video shows JX14 (MitoTrackerDeepRed-AF647 and CellMaskOrange-AF546) and NHA (MitoTrackerGreen-AF488 and CellMaskOrange-AF546) forming, extending, and retracting TNTs under DMSO-vehicle conditions, 2µm scale bar.

**Supplemental Video SV3: Live Cell Confocal Time-lapse Depicting MED2-treated TNT Formation, Extension, and Retraction from JX14 to NHA.** High-resolution confocal image frames captured every three minutes using NIS Elements. Sequential images were processed in Imaris to show maximum intensity projection of TNT. Video shows JX14 (MitoTrackerDeepRed-AF647 and CellMaskOrange-AF546) forming, extending, and retracting deformed TNT to NHA (MitoTrackerGreen-AF488 and CellMaskOrange-AF546) under MED2-treatment conditions, 1µm scale bar.
