# Supplementary material for: MARCKS as a mediator and target for pathological tunneling nanotubes between patient-derived glioblastoma cells and astrocytes
Source: Cell Death Dis. 2026 Jul 13;17(1):867. doi: 10.1038/s41419-026-09109-x (PMC13642262; doi:10.1038/s41419-026-09109-x)
Supplement: Supplementary file 2 [file 41419_2026_9109_MOESM2_ESM.docx]

Supporting Information For:

**MARCKS as a Mediator and Target for Pathological Tunneling Nanotubes Between Patient Derived Glioblastoma Cells and Astrocytes**

Lauren C. Nassour-Caswell^1^, Mayada Ahmed^1^, Shane T. Rich-New^2^, Taylor L. Schanel^1^, Manoj Kumar^1^, Nicholas J. Eustace^3^, Timothy D. Rohrbach^4^, John S. Jarboe^5^, Patricia H. Hicks^1^, Joshua C. Anderson^1^, Christopher D. Willey^1*^

^1^Department of Radiation Oncology, Heersink School of Medicine, University of Alabama at Birmingham, Birmingham, AL 35249, USA.

^2^Department of Biochemistry and Molecular Biology, University of Alabama at Birmingham, Birmingham, AL 35233, USA

^3^Department of Radiation Oncology, City of Hope National Medical Center, Duarte, CA, 91010, USA

^4^Department of Natural Sciences and Mathematics, Richard Bland College of William & Mary, South Prince George, VA 23805, USA

^5^Central Alabama Radiation Oncology, Montgomery, AL 36106, USA.

*Corresponding Author Information

Christopher D. Willey, M.D., Ph.D.

Hale-Stephens ROAR Endowed Professor

Vice Chair of Research

Department of Radiation Oncology

The University of Alabama at Birmingham

619 19^th^ St S.

HSROC 2220B

Birmingham, AL 35249

Phone: 205.934.5670

Fax: 205.975.0784

[cwilley@uabmc.edu](mailto:cwilley@uabmc.edu)

**This file includes:**

Appendix 1: Supplemental Methods

Appendix 2: Supplemental Figure S1

Appendix 3: Supplemental Figure S2

Appendix 4: Supplemental Video SV1

Appendix 5: Supplemental Video SV2

Appendix 6: Supplemental Video SV3

**Appendix 1: Supplemental Methods**

**Representative confocal images in figures**

- [In FIJI] Z-stack ND2 files were imported into FIJI. Individual slices of image were Z-projected using maximum intensity projection. Brightness was autoscaled and scale bars were added manually, calibrated using a known distance. Individual fluorescent channels and merged images were exported as JPEG files for importing into PowerPoint.
- [In PowerPoint] JPEG images were imported and brightness was adjusted by +20%.
- [In IMARIS] Z-stack ND2 files were imported and converted using the IMARIS converter. Images were then processed using deconvolution (Richardson-Lucy, 40 iterations) and normal shading correction prior to visualization in volume view. Different orientations of the still image were created by manually moving the image to show desired views using Bitplane Imaris 10.1 Analysis Tool. The file was saved as an MP4 file.

**TNT identification and measurement in confocal images**

- [In ICY] Z-stack ND2 files with blinded file names were imported into the ICY Bioimage Opensource Manual TNT Annotation Plugin. The bottom slices were excluded from the analysis so only projections suspended above the substratum would be counted as TNTs. TNTs visible by contrast were manually identified, and the tracking tool was used to annotate individual TNTs and TNT-connected cells.
- [In FIJI] TNTs identified in ICY were manually measured in FIJI using the line tool. TNTs with diameters above 700nm were excluded. Numerical measurements were exported for downstream analysis in GraphPad Prism v10.1.

**Co-expression analysis in confocal images**

- [In FIJI] Z-stack ND2 files were imported into FIJI and Z-projected using maximum intensity projection. Images were autoscaled and background subtraction was applied. Intensity profiles for each fluorescent channel were generated using line or ROI analysis, and numerical values were exported for correlation analysis in GraphPad Prism v10.1.

**Image total cell counts in confocal images**

- [In FIJI] Z-stack ND2 files were imported into FIJI and the channel identifying individual cells stained with DAPI was selected. Images were Z-projected using maximum intensity projection, followed by background subtraction, median filtering, binary conversion, and despeckling. Cell quantification was performed using the 3D OC Object Counter with thresholds set to 100 and size exclusion parameters of 200–5000 µm². Object visualization and statistical outputs were generated for analysis in GraphPad Prism v10.1.

**Mitochondrial Quantification**

- [Cytation 5 imaging / Gen5 analysis software] NHAs were individually stained with whole-cell tracker blue, while JX14 cells or U87 cells were individually stained with MitoTrackerRed as described in the methods. Subpopulations of NHAs staining positive for both DAPI and Texas Red were identified and quantified. NHA-uptake of GBM-derived mitochondria values were normalized at each timepoint to the timepoint 0 control for analysis of high-throughput mitochondrial quantification in GraphPad Prism v10.1.
- [Confocal imaging / IMARIS analysis software] Z-stack ND2 files were imported and converted using the IMARIS converter. Nuclear surfaces were generated from the DAPI channel using a surface detail setting of 0.414 µm. Machine learning segmentation was applied across all channels, and objects were split using a seed point diameter corresponding to the smallest nucleus within the sample. Statistical outputs were collected, including mean intensity values for the mitochondrial channel (AF546). Surface creation parameters were saved and applied in batch processing for subsequent images, with manual adjustment of surface wrapping performed as needed. Values were exported and used for analysis of high-resolution mitochondrial quantification in GraphPad Prism v10.1.

**Appendix 2: Supplemental Figure S1**

**Supplemental Figure S1: Homotypic TNT Dynamics in JX14.** A) Representative maximum-intensity widefield fluorescent images of JX14 cells under different PKC-targeting drug conditions. B) Average TNT-connected cells **p<0.01 (VEH vs PMA+CytoB, actual p=0.0031), *p<0.05 (VEH vs EZ, actual p=0.0293) by one-way ANOVA with Dunnett’s comparison. C) Average TNTs per cell. D) Average TNT length **p<0.01 (VEH vs PMA +CytoB, actual p=0.0072), *p<0.05 (VEH vs PMA, actual p=0.0285 by one-way ANOVA with Dunnett’s comparison. E) Percentage of JX14 with JX14-derived mitochondria quantified longitudinally every 30min over 18h ****p<0.0001 (2h Peak, VEH vs PMA, actual p<0.0001, VEH vs EZ, actual p<0.0001, VEH vs PMA+CytoB, actual p<0.0001) by two-way ANOVA with repeated measures and Šidák’s comparison. N = 4 VEH, N = 3 PMA, N = 3 EZ, N = 3 PMA + CytoB were analyzed per experimental condition.

**Appendix 3: Supplemental Figure S2**

**Supplemental Figure S2: Proposed MARCKS-related TNT mechanism.** MARCKS is typically bound to the PIP_2_ at the cell membrane. Upon phosphorylation by PKC, MARCKS is released from PIP_2_ and TNT protrusions result from the dissociation. MARCKS may also be crosslinking necessary actin at the cell membrane in preparation for TNT protrusions. Made in Biorender.com.

**Appendix 4: Supplemental Video SV1**

**Supplemental Video SV1: Three-Dimensional View of TNT Suspended Above the Substratum Between NHA and JX14 Under Control Conditions.** High-resolution confocal image processed in Imaris to show maximum intensity projection and 3D orientation of TNT. Video shows JX14 (MitoTrackerRed-AF546) and NHA (CellTrackerBlue) connected by TNT positive for F-actin (AF488) containing MARCKS (anti-MARCKS-AF647), and JX14-derived mitochondria.

**Appendix 5: Supplemental Video SV2**

**Supplemental Video SV2: Live Cell Confocal Time-lapse Depicting Transient TNT Formation, Extension, and Retraction between NHA and JX14.** High-resolution confocal image frames captured every three minutes using NIS Elements. Sequential images were processed in Imaris to show maximum intensity projection of TNT. Video shows JX14 (MitoTrackerDeepRed-AF647 and CellMaskOrange-AF546) and NHA (MitoTrackerGreen-AF488 and CellMaskOrange-AF546) forming, extending, and retracting TNTs under DMSO-vehicle conditions, 2µm scale bar.

**Appendix 6: Supplemental Video SV3**

**Supplemental Video SV3: Live Cell Confocal Time-lapse Depicting MED2-treated TNT Formation, Extension, and Retraction from JX14.** High-resolution confocal image frames captured every three minutes using NIS Elements. Sequential images were processed in Imaris to show maximum intensity projection of TNT. Video shows JX14 (MitoTrackerDeepRed-AF647 and CellMaskOrange-AF546) forming, extending, and retracting deformed TNT to NHA (MitoTrackerGreen-AF488 and CellMaskOrange-AF546) under MED2-treatment conditions, 1µm scale bar.
